# Supplementary material for: Full-length transcriptome reconstruction reveals a large diversity of RNA and protein isoforms in rat hippocampus
Source: Nat Commun. 2019 Nov 1;10:5009. doi: 10.1038/s41467-019-13037-0 (PMC6825209; doi:10.1038/s41467-019-13037-0)
Supplement: Supplementary file 1 — Supplementary Information [file 41467_2019_13037_MOESM1_ESM.pdf]

Supplementary Information

Full-length transcriptome reconstruction reveals a large diversity of RNA and protein isoforms in rat hippocampus

Xi Wang[1,2,#,*], Xintian You[1,3,#], Julian D. Langer[4], Jingyi Hou[1], Fiona Rupprecht[4], Irena Vlatkovic[4], Claudia Quedenau[1], Georgi Tushev[4], Irina Epstein[4], Bernhard Schaefke[5,6], Wei Sun[5], Liang Fang[5,6], Guipeng Li[5,6], Yuhui Hu[5], Erin M. Schuman[4], Wei Chen[5,6,*]

1 Max Delbrück Center for Molecular Medicine, Berlin, Germany
2 German Cancer Research Center, Heidelberg, Germany
3 Max Planck Institute for Molecular Genetics, Berlin, Germany
4 Max Planck Institute for Brain Research, Frankfurt, Germany
5 Department of Biology, Southern University of Science and Technology, Shenzhen, China
6 Medi-X Institute, SUSTech Academy for Advanced Interdisciplinary Studies, Southern University of Science and Technology, Shenzhen, China

# These authors contributed equally: X.W. and X.Y.
* Corresponding authors: X.W. xi.wang@dkfz.de and W.C. chenw@sustech.edu.cn

CONTENTS

Supplementary Figures 1-9 …………………………………… Page 2
Supplementary Tables 1-2 …………………………………… Page 13

Supplementary Figure 1

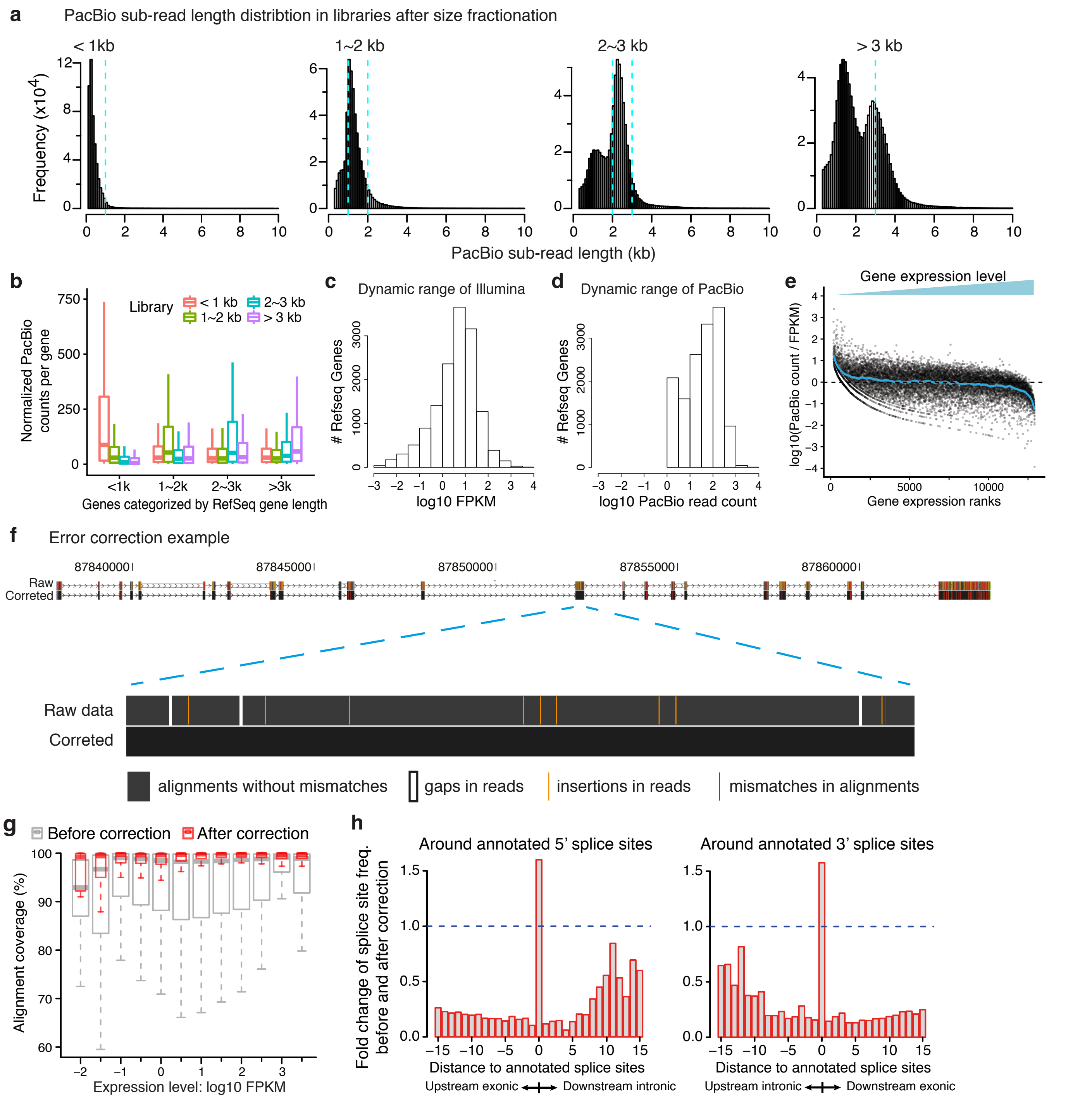

Supplementary Figure 1 | Size fractionation, cDNA normalization and error correction in the hybrid sequencing workflow.
a. Histograms show the distribution of PacBio read length in the four libraries fractionated by cDNA size. From left to right, the expected size ranges are: <1kb, 1~2kb, 2~3kb, and >3kb. **b**. Boxplots show the distribution of normalized PacBio read counts per gene derived from genes of different length range in the four size-fractionated libraries. In all four libraries, there were more PacBio reads derived from genes of expected length. **c**. Histogram shows that the gene expression dynamic range in rat hippocampus spanning seven orders of magnitude, as measured by the Illumina sequencing of the non-normalized library. **d**. Histogram shows that the number of PacBio reads per gene spanned three orders of magnitude, considerably decreased from the true gene expression dynamic range in rat hippocampus. **e**. Similar to Figure 1b, but the ratio of PacBio read count over gene expression level estimated by Illumina sequencing data was plotted against gene expression ranks. Each dot represents a gene, and the smoothed curve representing the overall trend is shown in blue. **f**. An example shows the performance of error correction. **g**. The alignment coverage (i.e. the ratio of alignment length over read length) before and after error correction was plotted against gene expression levels. **h**. Alignment precision at canonical splice sites was increased after error correction. **b**,**g**. Box edges represent quartiles, whiskers represent extreme data points no more than 1.5 times the interquartile range. Source data for panels **c**-**e** are provided in a Source Data file.

Supplementary Figure 2

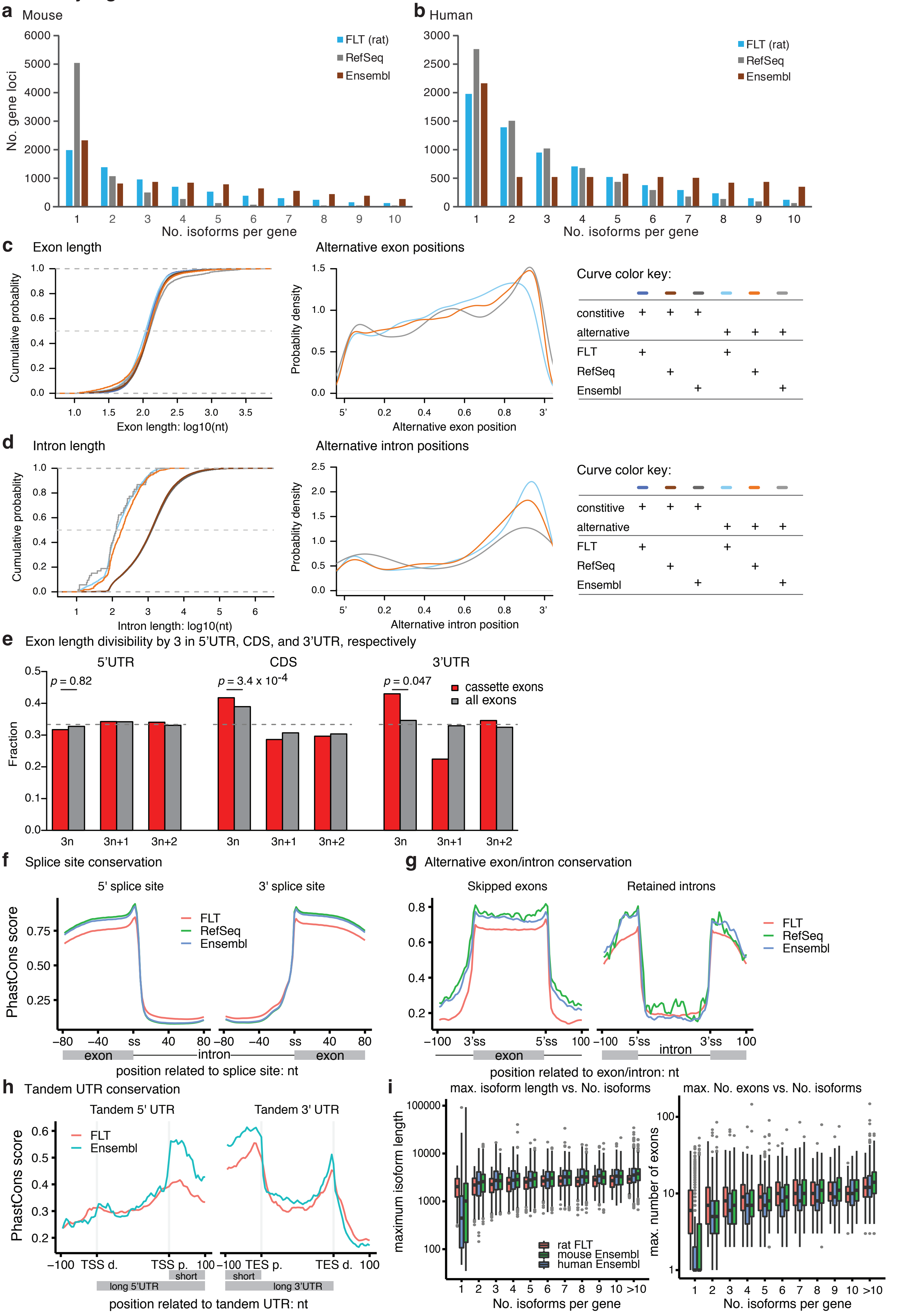

(see the legend on the next page)

Supplementary Figure 2 | Features of our FLT collection.

a. Similar to Figure 3a, but the number of isoforms per gene in our FLT was compared to mouse RefSeq and Ensembl annotation. **b**. Similar to Figure 3a, but the number of isoforms per gene in our FLT was compared to human RefSeq and Ensembl annotation. **c**. The distribution of (alternative) exon length, alternative exon positions in our FLT compared to rat RefSeq and Ensembl annotation. **d**. The distribution of (alternative) intron length, alternative intron positions in our FLT compared to rat RefSeq and Ensembl annotation. **e.** The divisibility by 3 of the exon length in 5′UTR, CDS, and 3′UTR. In CDS, there was a significant enrichment of 3-divisibile cassette exons (Fisher's exact test). **f**. The PhastCons scores at splicing sites in our FLT compared to that in rat RefSeq and Ensembl annotation. **g**. The PhastCons scores on skipped exons/retained introns and their flanking regions in our FLT compared to that in rat RefSeq and Ensembl annotation. **h**. The PhastCons scores on tandem UTRs in our FLT compared to that in rat RefSeq and Ensembl annotation. **i**. In our FLT as well as in mouse and human Ensembl annotation, the number of isoforms per gene was positively correlated with the maximum transcript length and the maximum number of exons. Box edges represent quartiles, whiskers represent extreme data points no more than 1.5 times the interquartile range.

Supplementary Figure 3

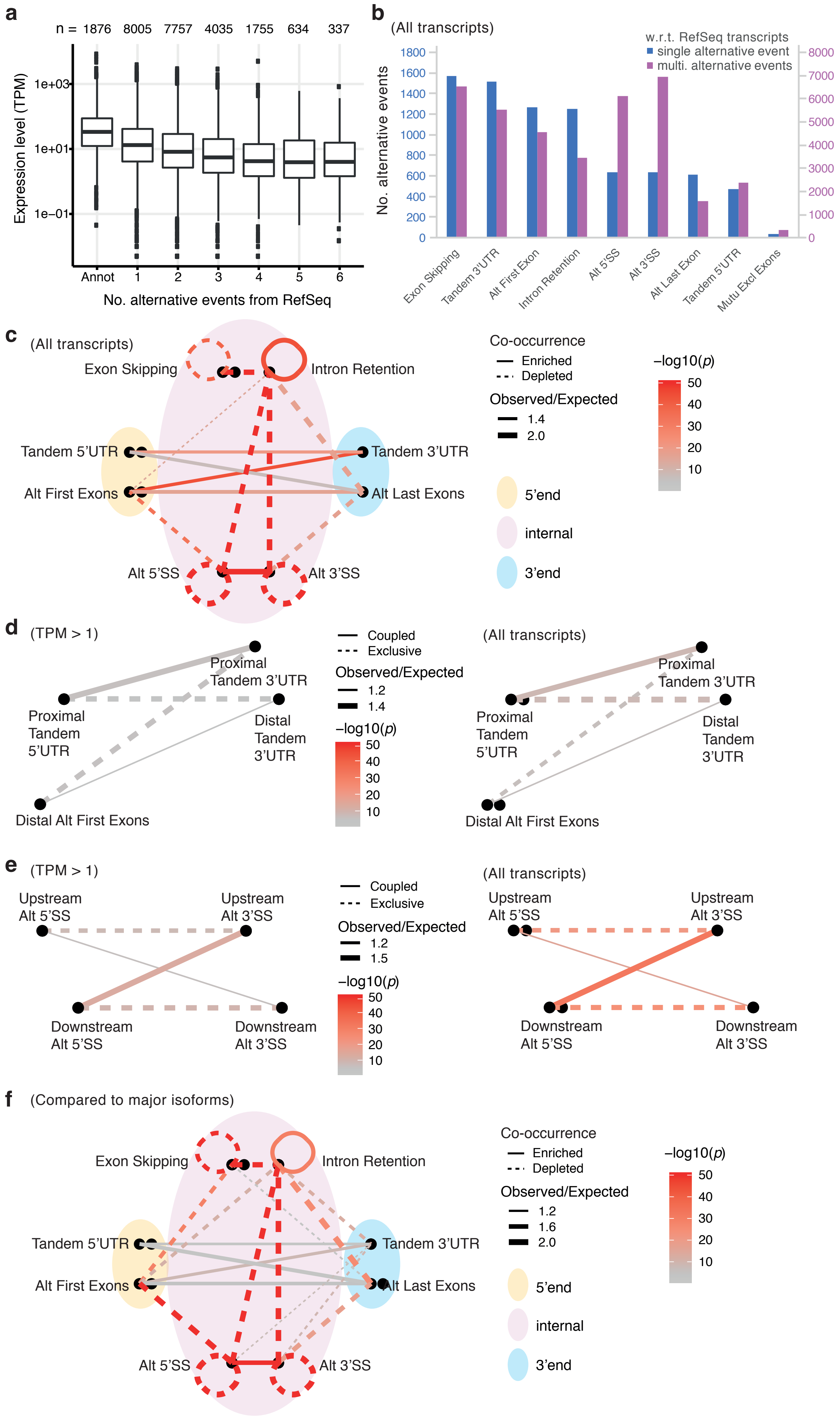

(see the legend on the next page)

Supplementary Figure 3 | Transcript isoform diversity and co-occurrence of alternative RNA processing events.

a. Isoform expression level was plotted against the number of alterative events derived from the closest RefSeq transcripts. The annotated isoforms in general had higher expression levels. Box edges represent quartiles, whiskers represent extreme data points no more than 1.5 times the interquartile range. **b.** Similar to Figure 3c, but all isoforms in our FLT were considered. **c**. Similar to Figure 3d, but all isoforms in our FLT were considered. **d**. Separating the co-occurrence of alternative events at the transcript ends into distal and proximal ones. Shown were isoforms with TPM > 1 (left) and all isoforms (right). **e**. Separating the co-occurrence of alternative 5′ and 3′ splice sites into upstream and downstream ones. Shown were isoforms with TPM > 1 (left) and all isoforms (right). **f**. Similar to Figure 3c, but all isoforms were compared to the major isoforms in the FLT for analyzing alternative events. **c**-**f**. Line type: enrichment or depletion of the co-occurrence; line width: the ratio between observed co-occurrence and expected co-occurrence; line color: -log10(P values) of the enrichment or depletion (one-tailed binomial tests).

Supplementary Figure 4

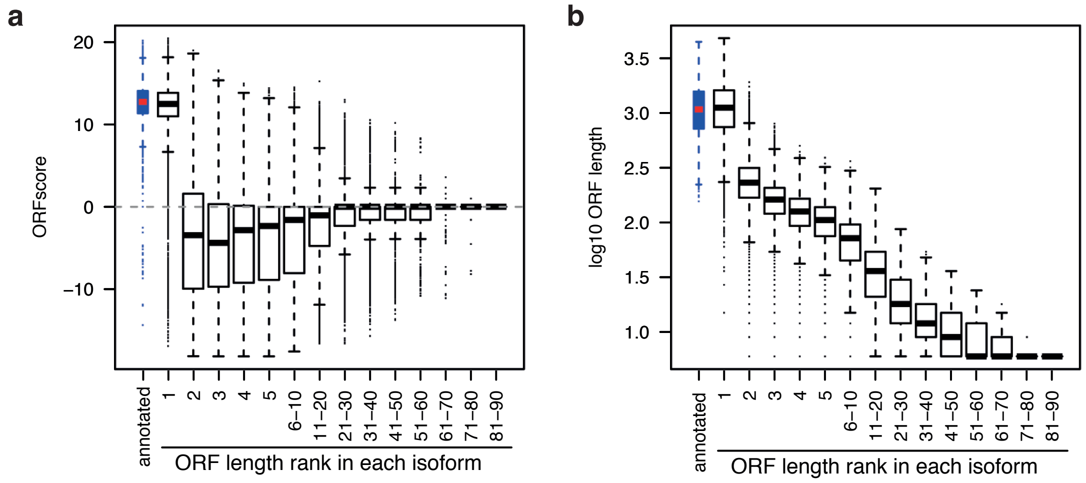

Supplementary Figure 4 | Characteristics of ORFscores and ORF length in translationally active isoforms.

a. Boxplots show the distribution of ORFscores of all possible ORFs binned by the rank of ORF length in each isoform, compared to distribution of the annotated ORFs. **b**. Boxplots show the distribution of ORF length of all possible ORFs binned by the rank of ORF length in each isoform, compared to distribution of the annotated ORFs. Box edges represent quartiles, whiskers represent extreme data points no more than 1.5 times the interquartile range.

Supplementary Figure 5

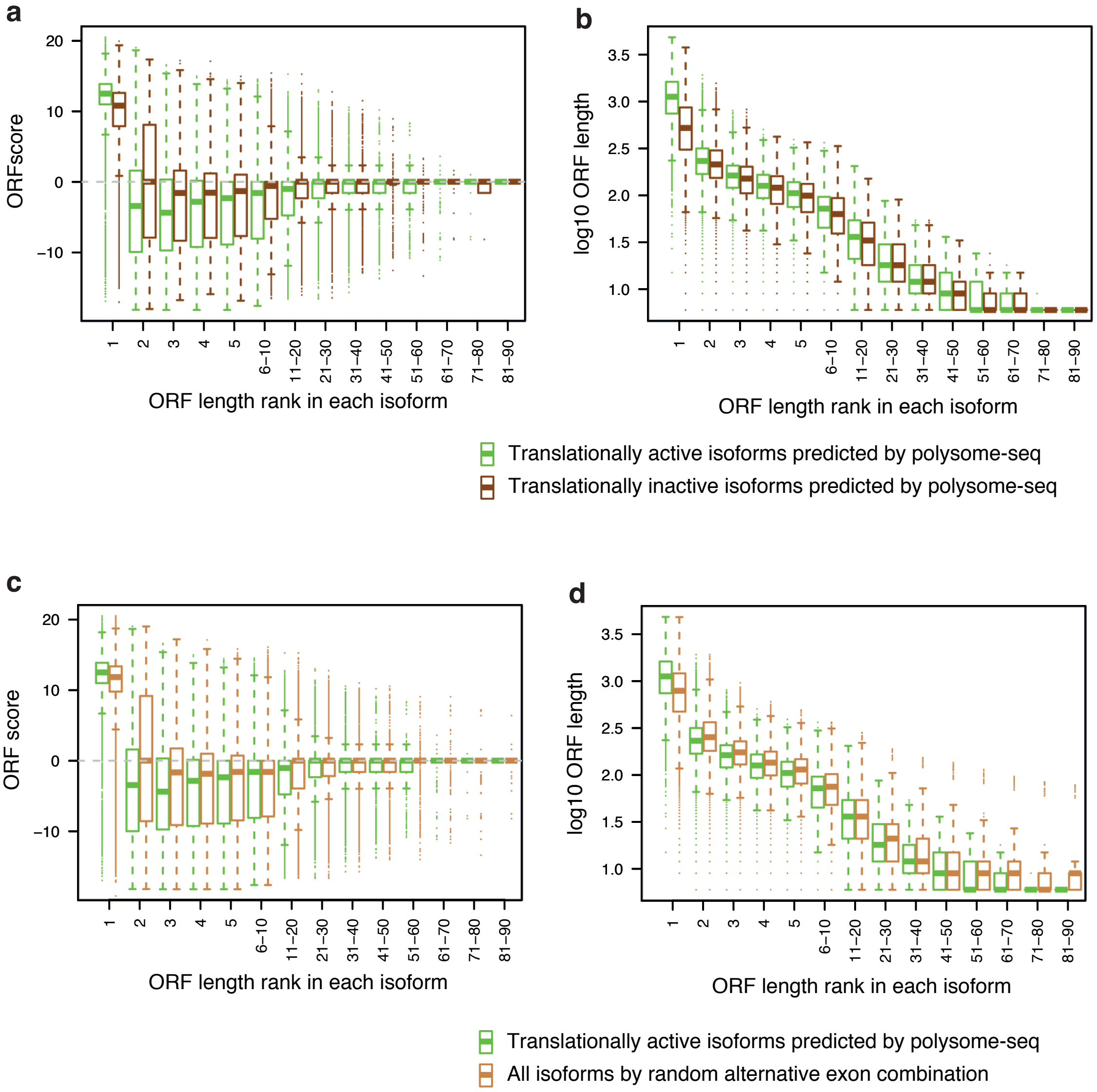

Supplementary Figure 5 | Characteristics of ORFscores and ORF length in translationally inactive isoforms and randomly generated isoforms.
a. Boxplots show the distribution of ORFscores of all possible ORFs binned by the rank of ORF length in each isoform, comparing between translationally active and inactive isoforms. **b**. Boxplots show the distribution of ORF length of all possible ORFs binned by the rank of ORF length in each isoform, comparing between translationally active and inactive isoforms. **c**. Similar to **a**, but comparing between translationally active isoforms and randomly constructed isoforms. **d**. Similar to **b**, but comparing between translationally active isoforms and randomly constructed isoforms. Box edges represent quartiles, whiskers represent extreme data points no more than 1.5 times the interquartile range.

Supplementary Figure 6

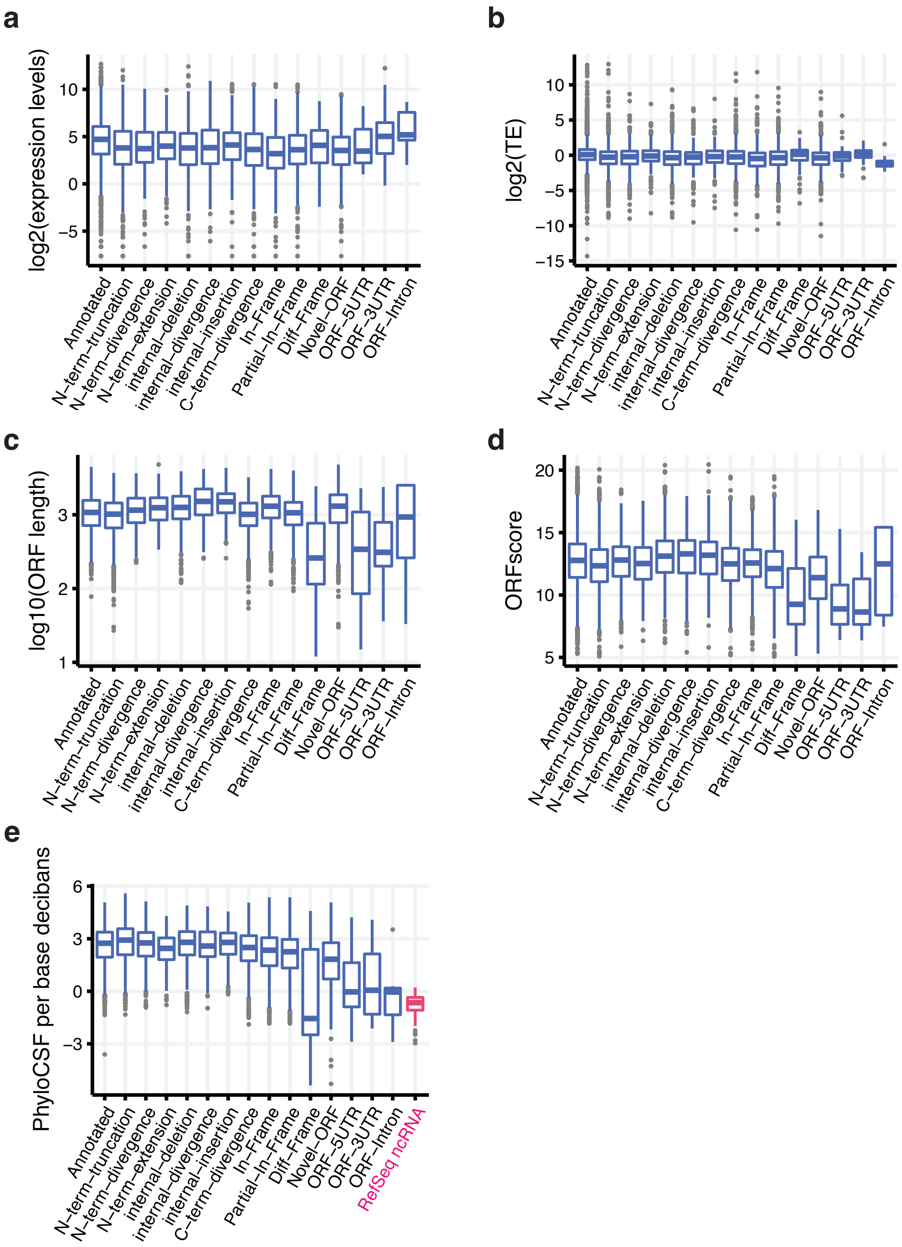

Supplementary Figure 6 | Characteristics of different ORF types.
a. Boxplots compare the expression levels of ORFs in different types. **b**. Boxplots compare the translational efficiency of ORFs in different types. **c**. Boxplots compare the length of ORFs in different types. **d**. Boxplots compare the ORFscores of ORFs in different types. **e**. Boxplots compare the PhyloCSF of ORFs in different types, as well as the annotated ncRNAs in RefSeq (red). Box edges represent quartiles, whiskers represent extreme data points no more than 1.5 times the interquartile range.

Supplementary Figure 7

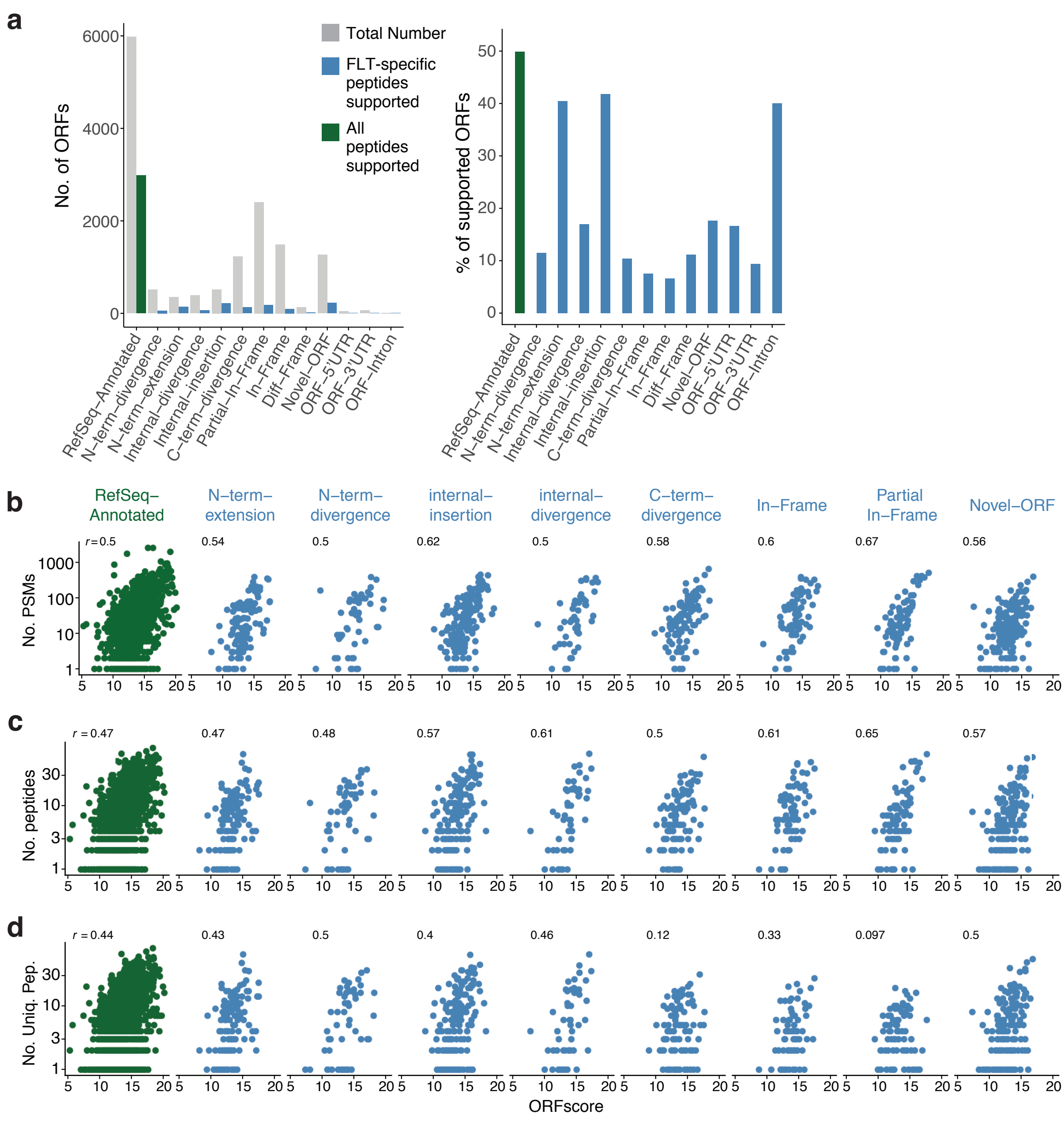

Supplementary Figure 7 | Characteristics of ORFs validated by MS-based proteomics data. **a**. Similar to Figure 6a, but the FDR threshold at 0.01 was based on the estimation using only the FLT database. **b**. The number of PSMs, c. the number of peptides, and **d**. the number of unique peptides were plotted against the ORFscores in each ORF category. Except for the RefSeq-annotated ORFs where all peptides were used, in the other ORF categories only the FLT-specific peptides were used. Pearson's correlation coefficients are indicated in each plot at the top left.

Supplementary Figure 8

Rpp14
Novel ORF: FLT5062, FLT5063

MLKMWSGLQRRLWQHRVVPTGQCFRCVHMKVGDRAELSRAFTQQD
VATFSELTGDANPLHLSEDFAKHTRFGKTVVHGVLINGLISALLGTKM
PGPGCVFLSQEIKFPAPLYIGEVVLASAEVKRLKQSVAVVEVSCCVIES
KKTVMEGLVKIMVPGAPRS

Nudt13
Novel ORF: FLT5388, FLT5389, FLT5390

MAHQKRQLVPATMETEARASSLLPQQPRQHTRHCHLRTTQTLQGGGL
TDGTLCEELREGADAGVWV

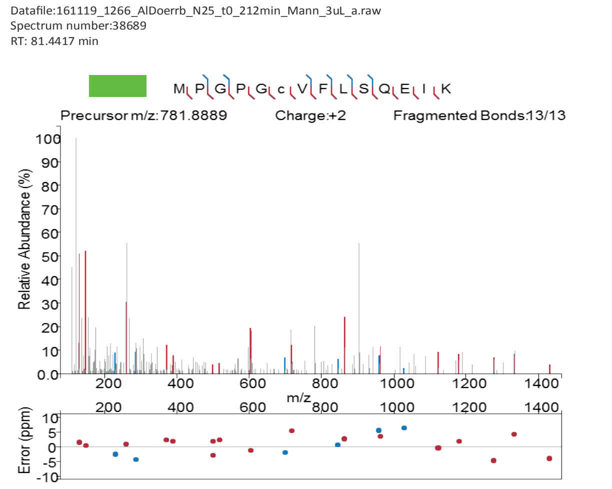

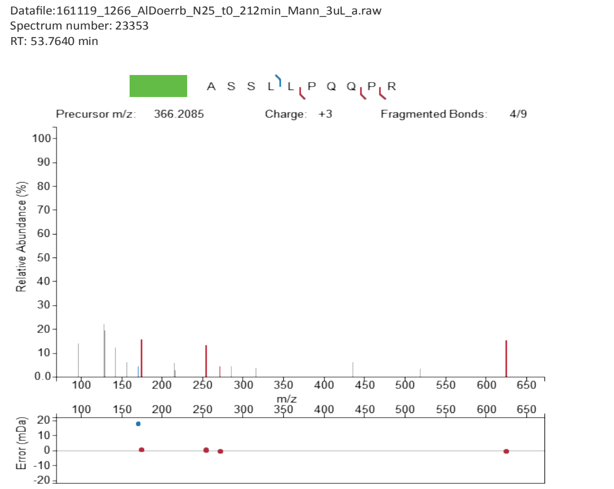

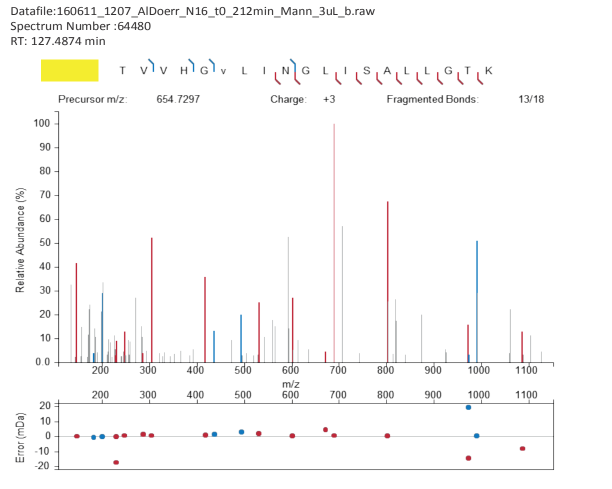

RefSeq ORF

MSLYCGTFFRRKSFGCYRLLSTYVTKARYLFELKEDDEACRKAQQTGL
FYLFHDLDPLLQESGHRYLVPRLSRAELEGLLGKFGQDSQRIEDSVLVG
CSNEQEAWFALDLGLKSASSVRASLPKSEMEAELGGSFVKLRQALLQL
NSVDSSLLFTAQALLRWHDGHQFCSKSGQPTQKNMAGSKRVCPSNNII
YYPQMAPVVITLVSDGARCLLARQSSFPRGLYSALAGFCDIGERVEEAV
HREVAEEVGLEVENIQYSASQHWPFPNSSLMIACHATVKPGHTEIQVNL
KELEAAAWFSLDEVATALRRKGSFAQQQREASPLMLPPKLAVAHHMI
KEWVEKQSRSSLAA

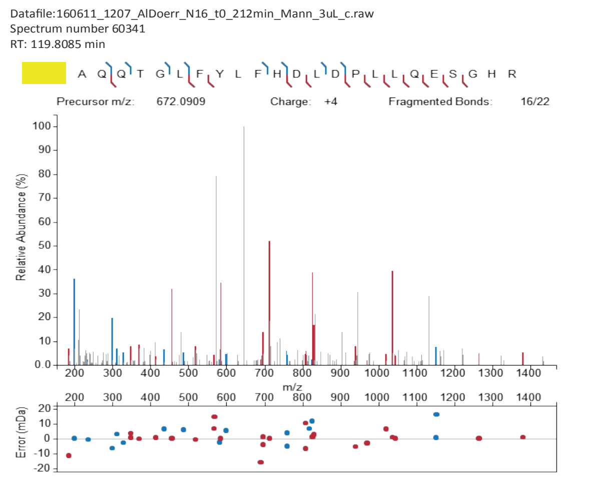

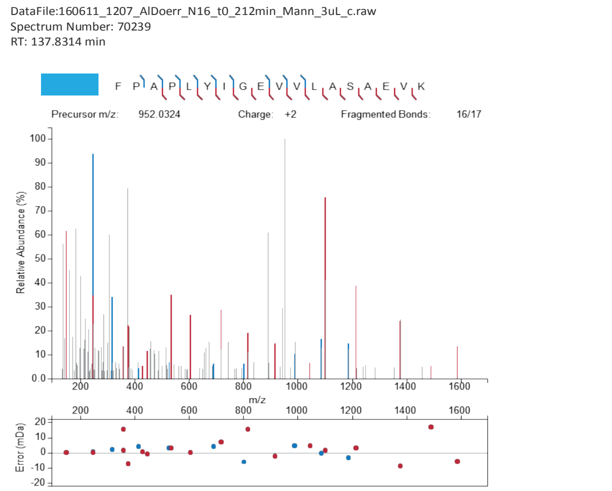

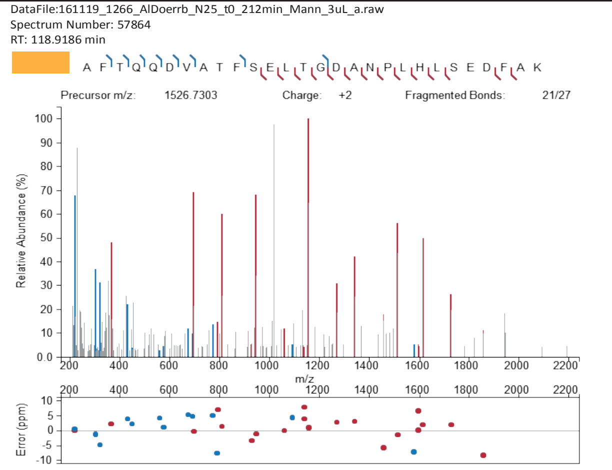

Supplementary Figure 8 | Annotated spectra for the peptides presented in Fig. 6c,d.

Supplementary Figure 9

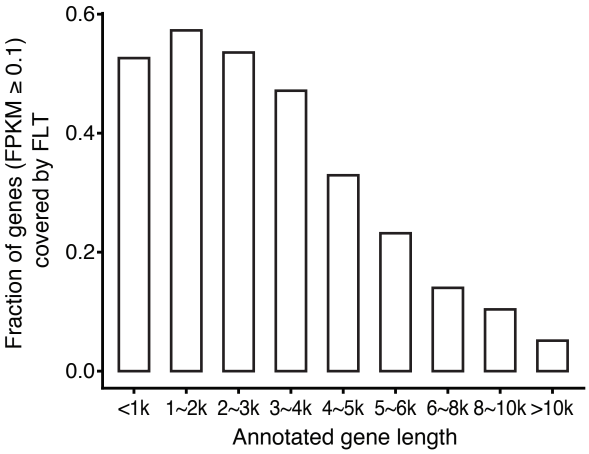

Supplementary Figure 9 | The percentage of rat genes expressed (≥ 0.1 FPKM) in hippocampus covered by our FLT across different gene length.

Supplementary Table 1 | PacBio sequencing data summary.

Libraries	<1kb	1~2kb	2~3kb	≥3kb	Total
# SMRT cells	23	21	20	31	95
# raw reads	568,636	777,440	1,034,557	1,694,533	4,075,116

Supplementary Table 2 | List of primers used in this study.

PacBio library preparation

**Name**	**Sequence (5′ --> 3′)**
SMRT-PCR	AAGCAGTGGTATCAACGCAGAGTAC

5′CAGE

**Name**	**Sequence (5′ --> 3′)**
N15-oligo	TACACGACGCTCTTCCGATCTNNNNNNNNNNNNNNN
cap GN5 up*	CAGACGTGTGCTCTTCCGATCTGNNNNN-P
cap N6 up*	CAGACGTGTGCTCTTCCGATCTNNNNNN-P
cap 5′adaptor down*	P-AGATCGGAAGAGCACACGTCTG-NH2
cap forward primer	AATGATACGGCGACCACCGAGATCTACACTCTTTCCCTACACGACGCTCTTCCGATC
cap reverse primer**	GATCGGAAGAGCACACGTCTGAACTCCAGTCACNNNNNNATCTCGTATGCCGTCTTCTGCTTG

* cap GN5 up / cap N6 up and cap 5′ adaptor down form double stranded 5′ linkers

** the underlined N(6) comprises sample multiplex barcodes
