# Supplementary material for: Full-length transcriptome reconstruction reveals a large diversity of RNA and protein isoforms in rat hippocampus
Source: Nat Commun. 2019 Nov 1;10:5009. doi: 10.1038/s41467-019-13037-0 (PMC6825209; doi:10.1038/s41467-019-13037-0)
Supplement: Supplementary file 3 — Description of Additional Supplementary Files [file 41467_2019_13037_MOESM3_ESM.docx]

**Description of Supplementary Files**

**File Name:** Supplementary Data 1

**Description:** ORFs with loss or gain of signal peptides due to extended, divergent, or truncated N-terminus.

**File Name:** Supplementary Data 2

**Description:** Conserved novel ORFs identified in the known gene loci.

**File Name:** Supplementary Data 3

**Description:** Identified peptides matching FLT-specific ORFs.
