# Supplementary material for: Full-length transcriptome reconstruction reveals a large diversity of RNA and protein isoforms in rat hippocampus
Source: Nat Commun. 2019 Nov 1;10:5009. doi: 10.1038/s41467-019-13037-0 (PMC6825209; doi:10.1038/s41467-019-13037-0)
Supplement: Supplementary file 7 — Reporting Summary [file 41467_2019_13037_MOESM7_ESM.pdf]

nature research

Corresponding author(s): Xi Wang, Wei Chen

Last updated by author(s): Oct 15, 2019

Reporting Summary

Nature Research wishes to improve the reproducibility of the work that we publish. This form provides structure for consistency and transparency in reporting. For further information on Nature Research policies, see Authors & Referees and the Editorial Policy Checklist .

Statistics

For all statistical analyses, confirm that the following items are present in the figure legend, table legend, main text, or Methods section.

n/a	Confirmed	
☐	☒	The exact sample size (*n*) for each experimental group/condition, given as a discrete number and unit of measurement
☒	☐	A statement on whether measurements were taken from distinct samples or whether the same sample was measured repeatedly
☐	☒	The statistical test(s) used AND whether they are one- or two-sided<br>*Only common tests should be described solely by name; describe more complex techniques in the Methods section.*
☒	☐	A description of all covariates tested
☐	☒	A description of any assumptions or corrections, such as tests of normality and adjustment for multiple comparisons
☐	☒	A full description of the statistical parameters including central tendency (e.g. means) or other basic estimates (e.g. regression coefficient) AND variation (e.g. standard deviation) or associated estimates of uncertainty (e.g. confidence intervals)
☐	☒	For null hypothesis testing, the test statistic (e.g. *F*, *t*, *r*) with confidence intervals, effect sizes, degrees of freedom and *P* value noted<br>*Give P values as exact values whenever suitable.*
☒	☐	For Bayesian analysis, information on the choice of priors and Markov chain Monte Carlo settings
☒	☐	For hierarchical and complex designs, identification of the appropriate level for tests and full reporting of outcomes
☒	☐	Estimates of effect sizes (e.g. Cohen's *d*, Pearson's *r*), indicating how they were calculated

Our web collection on statistics for biologists contains articles on many of the points above.

Software and code

Policy information about availability of computer code

Data collection	PacBio Sequencing: SMRT-Portal analysis suite v2.2.0<br>Illumina Sequencing: Illumina OLB1.9 or CASAVA1.8 software
Data analysis	Flexbar (v2.4) used for adapter trimming<br>Bowtie2 (v2.1.0)/Tophat2(v2.0.10) used to map the reads<br>RSEM (v1.3.0) used to estimate the isoform expression level<br>GMAP (version 2016-09-23) to align corrected PacBio reads against the rat reference genome<br>proovread (v2.13.10) used to correct PacBio sequencing errors: https://github.com/BioInf-Wuerzburg/proovread<br>IPEC for Illumina-based PacBio error correction, available at https://github.com/arthuryxt/IPEC<br>FuLeTA for full-length transcriptome analysis available at https://github.com/sunlightwang/FuLeTA<br>Thermo Proteome DiscovererTM (PD) software suite (version 2.3) used for peptide matching<br>R (>3.4.0), Perl (v5.16.3), and bash (4.2.46) used to process data and perform statistical analysis with custom scripts

For manuscripts utilizing custom algorithms or software that are central to the research but not yet described in published literature, software must be made available to editors/reviewers. We strongly encourage code deposition in a community repository (e.g. GitHub). See the Nature Research guidelines for submitting code & software for further information.

Data

Policy information about availability of data

All manuscripts must include a data availability statement. This statement should provide the following information, where applicable:

- Accession codes, unique identifiers, or web links for publicly available datasets
- A list of figures that have associated raw data
- A description of any restrictions on data availability

All the raw sequencing data along with processed data are deposited into NCBI GEO database with accession number GSE128136.
The MS-based proteomics data are available via PRIDE with accession number PXD008596 ("untreated/t0" data files).

Field-specific reporting

Please select the one below that is the best fit for your research. If you are not sure, read the appropriate sections before making your selection.

☒ Life sciences ☐ Behavioural & social sciences ☐ Ecological, evolutionary & environmental sciences

For a reference copy of the document with all sections, see nature.com/documents/nr-reporting-summary-flat.pdf

Life sciences study design

All studies must disclose on these points even when the disclosure is negative.

Sample size	Experiments including total RNA-seq, RNA-seq for compartmentalized RNA, polysome profiling, ribosome footprinting were performed with at least 2 replicates. For each replicate, rats might be pooled due to limited materials obtained from one rat for sequencing library preparation.
Data exclusions	No data exclusions.
Replication	All attempts at replication were successful.
Randomization	n/a, all experiments were performed with four-week-old male rats at the normal condition.
Blinding	n/a, all experiments were performed with four-week-old male rats at the normal condition.

Reporting for specific materials, systems and methods

We require information from authors about some types of materials, experimental systems and methods used in many studies. Here, indicate whether each material, system or method listed is relevant to your study. If you are not sure if a list item applies to your research, read the appropriate section before selecting a response.

Materials & experimental systems

n/a	Involved in the study	
☒	☐	Antibodies
☒	☐	Eukaryotic cell lines
☒	☐	Palaeontology
☐	☒	Animals and other organisms
☒	☐	Human research participants
☒	☐	Clinical data

Methods

n/a	Involved in the study	
☒	☐	ChIP-seq
☒	☐	Flow cytometry
☒	☐	MRI-based neuroimaging

Animals and other organisms

Policy information about studies involving animals; ARRIVE guidelines recommended for reporting animal research

Laboratory animals	Sprague Dawley rats, male, four-week old, housed in standard cages and fed standard lab chow and water ad libitum
Wild animals	n/a
Field-collected samples	n/a
Ethics oversight	The procedures involving animal treatment and care were conducted in conformity with the institutional guidelines that are in compliance with national and international laws and policies (DIRECTIVE 2010/63/EU; German animal welfare law; FELASA guidelines). The animals were euthanized according to annex 2 of § 2 Abs. 2 Tierschutz-Versuchstier-Verordnung.

Note that full information on the approval of the study protocol must also be provided in the manuscript.
